# Intestinal drug transporters in pathological states: an overview

**DOI:** 10.1007/s43440-020-00139-6

**Published:** 2020-07-27

**Authors:** Marek Drozdzik, Izabela Czekawy, Stefan Oswald, Agnieszka Drozdzik

**Affiliations:** 1 Department of Pharmacology, Pomeranian Medical University, Powstancow Wlkp 72, 70-111 Szczecin, Poland; 2 Department of Pharmacology, Medicine University Greifswald, Friedrich-Ludwig-Jahn-Straße 17, 17489 Greifswald, Germany; 3 Institute of Pharmacology and Toxicology, Rostock University Medical Center, 18051 Rostock, Germany; 4 Department of Integrated Dentistry, Pomeranian Medical University, Powstancow Wlkp 72, 70-111 Szczecin, Poland

**Keywords:** Gastrointestinal pathology, General pathology, Drug transporters

## Abstract

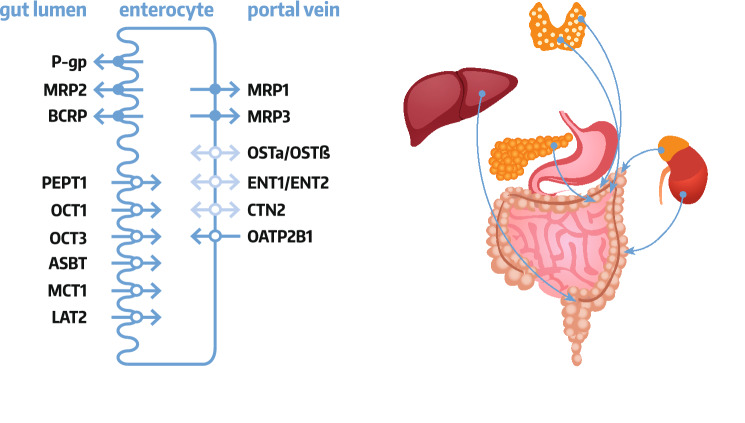

## Introduction

The gastrointestinal tract plays a crucial role in bioavailability determination of orally administered drugs, which may vary depending on, e.g., physicochemical characteristics of drug molecules (hydrophobicity, pKa, solubility) or specific drug formulation and function of the gastrointestinal tract, i.e., pH, water content, motility, drug metabolizing enzyme and drug transporters expression and activity as well as disease states.

Drug transporters constitute one of the most important factors governing drug movements across intestinal wall, and constitute a part of its functional barrier (along with phase I and II drug metabolizing enzymes). The transporters belong to transmembrane proteins that can be localized in both apical and basolateral membranes of enterocytes, and are engaged in cellular uptake or efflux processes of endogenous compounds (e.g., bile acids, sterols), nutrients (e.g., peptides) and xenobiotic compounds. The membrane transporters are classified into two major superfamilies, ATP-binding cassette transporters (ABC, consisting of about 50 members, which are subdivided into 7 families) and solute carriers (SLC, more than 400 membrane proteins grouped into over 60 families). Only few of the transporters, about 15–20, were characterized for their distinct role in drug transport [1]. Functionally, transporters can be classified into two principal classes: the ABC transporters (mediate extracellular efflux) and the SLC carriers (mediate cellular influx and/or cellular efflux). Related to their function, to the efflux transporters are classified: multidrug resistance family (MDR, ABCB), multidrug resistance protein family (MRP, ABCC), breast cancer resistance protein (BCRP, ABCG2), multidrug and toxin extrusion protein family (MATE, SLC47), whereas to the uptake carriers: organic anion transporting polypeptide family (OATP, SLC21/SLCO), organic anion transporter family (OAT, SLC22), organic cation transporter family (OCT, SLC22) or organic cation/carnitine transporter family (OCTN, SLC22). Coordinated function of the uptake and efflux transporters/carriers mediate vectorial transport of molecules across cell membranes (examples are presented in Table 1).

**Table 1:** Selected substrates of the transporters presented in the review

Transporter	Substrate
ABC transporter	
* ABCB1*/P-glycoprotein	Actinomycin D, aldosterone, amitriptyline, amprenavir, atorvastatin, β-amyloid, carbamazepine, celiprolol, chloropromazine, clopidogrel, citalopram, colchicine, corticosterone, cortisol, cyclosporine A, daunorubicin, dexamethasone, digoxin, diltiazem, doxycycline, doxorubicin, erythromycin, etoposide, fexofenadine, imatinib, indinavir, irinotecan, itraconazole, ketoconazole, lamotrigine, lansoprazole, levetiracetam, levofloxacin, loperamide, losartan, lovastatin, melphalan, methylprednisolone, mitomycin C, mitoxantrone, morphine, nelfinavir, omeprazole, ondansetron, paclitaxel, pantoprazole, pentazocine, phenobarbital, phenothiazine, phenytoin, propanolol, quinidine, ranitidine, rhodamine 123, rifampicin, ritonavir, saquinavir, simvastatin, sirolimus, sparfloxacin, tacrolimus, talinolol, 99mTc-MIBI, teniposide, terfenadine, tetracycline, topotecan, vecuronium, verapamil, vinblastine, vincristine
* ABCG2*/BCRP	Canertinib, cimetidine, gefitinib, glyburide, imatinib, irinotecan, lamivudine, methotrexate, mitoxantrone, nilotinib, nitrofurantoin, pantoprazole, prazosin, rosuvastatin, sulfasalazine, topotecan, urate
* ABCC1*/MRP1	Aflatoxin B1-epoxide-GS, bilirubin-G, cyclophosphamide-GS, difloxacin, doxorubicin-GS, estradiol 17βD-G, estrone-3-S, etacrynic acid-GS, etoposide-G, gefitinib, glutathione, grepafloxacin, hydroxynonenal-GS, hyodeoxycholate-G, imatinib, irinotecan, melphalan-GS, methotrexate, mitoxantrone, prostaglandin A2-GS, ritonavir, saquinavir, teniposide, topotecan, vinblastine, vincristine; sulfate conjugates (-S), glutathione conjugates (-GS), glucuronide conjugates (-G)
* ABCC2*/MRP2	Adefovir, aflatoxin B1-epoxide-GS, ampicillin, azithromycin, bilirubin-G, cefodezime, ceftriaxone, cidofovir, cisplatin, cyclophosphamide-GS, dinitrophenyl-GS, doxorubicin, doxorubicin-GS, epirubicin, estradiol 17βD-G, estrone-3-S, etacrynic acid-GS, etoposide-G, etoposide, glutathione, grepafloxacine, hydroxynonenal-GS, hyodeoxycholate-G, indinavir, irinotecan, lopinavir, melphalan-GS, methotrexate, mitoxantrone, nelfinavir, olmesartan, prostaglandin A2-GS, ritonavir, saquinavir, SN-38-G, temocaprilate, valsartan, vinblastine, vincristine; sulfate conjugates (-S), glutathione conjugates (-GS), glucuronide conjugates (-G)
* ABCC3*/MRP3	Acetaminophen-G, bile salts, clopidogrel, estradiol-17β-G, ethenyl estradiol, etoposide, fexofenadine, leukotriene C4, methotrexate, phytoestrogen conjugates, resveratrol conjugates, teniposide, vincristine; glucuronide conjugates (-G)
* ABCC4*/MRP4	6-Mercaptopurine, 6-thioguanine, acyclovir, adefovir, bile salts, cefazolin,ceftizoxime, cholate, conjugated steroids, folate, furosemide, glycocholate, hydrochlorothiazide, leucovorin, methotrexate, olmesartan, p-aminohippurate, para-methoxy-*N*-ethylamphetamine, ritonavir, taurocholate, tenofovir, topotecan, urate
SLC carrier	
* SLC10A2*/ASBT	Benzothiazepine, benzothiepene derivates, conjugated and unconjugated bile acids, dimeric bile acid analogues, taurocholic acid, naphtol derivates
* SLC15A1*/PEPT1	5-Aminolevulinic acid, carnosine, cefadroxil, cephalexin, D-Phe-Ala, D-Phe-Gln, glibenclamide, nateglinide, penicillin G (benzylpenicilline), valacyclovir
* SLC16A1*/MCT1	L-Lactic acid, nateglinide, pyruvic acid, salicylates, valproic acid, β-D-hydroxybutyric acid, γ-hydroxybutyric acid
* SLC22A1*/OCT1	5-Hydroxytryptamine, acyclovir, choline, desipramine, ganciclovir, metformin, metformin, oxaliplatin, PGE2, PGF2α
* SLC22A2*/OCT2	Acetylcholine, aflatoxin B1, amantadine, amiloride, berberine, bile acids, choline, cimetidine, cisplastin, creatinine, dopamine, D-tubocurarine, epinephrine, ethidium bromide, famotidine, guanidine, histamine, ifosfamide, lamivudine, memantine, metformin, norepinephrine, oxaliplatin, pancuronium, paraquat, pindolol, propranolol, ranitidine, serotonin, varenicline, zalcitabine
* SLC22A3*/OCT3	Acetylcholine, amantadine, atropine, choline, cimetidine, citalopram, clonidine, corticosterone, creatinine, D-amphetamine, desipramine, dopamine, epinephrine, etilefrine, granisetron, guanidine, histamine, imipramine, ketamine, L-carnitine, memantine, metformin, mitoxantrone, nicotine, norepinephrine, phencyclidine, prazosine, progesterone, ranitidine, serotonin, testosterone, tropisetron, verapamil
* SLC22A4*/OCTN1	Acetylcholine, carnitine, doxorubicin, entecavir, ergothioneine, gabapentin, imatinib, metformin, mitoxantrone, oxaliplatin, pregabalin, pyrilamine, quinidine, tiotropium ipratropium, verapamil
* SLC22A5*/OCTN2	Carnitine, cephaloridine, cephaloridine, emetine, entecavir, etoposide, imatinib, ipratropium, spironolactone, tiotropium, verapamil
* SLC22A6*/OAT1	Adefovir, cephaloridin, ciprofloxacin, cyclic nucleotides (cAMP, cGMP), folates, indoksyl sulfate, methotrexate, PGE2, PGF2α, pravastatin, uric acid, zidovudine
* SLC22A7*/OAT2	5-Fluorouracil, acyclovir, bumetadine, cGMP, creatinine, DHEAS, diclofenac, entenavir, estrogen sulphate, ganciclovir, irinotecan, PAH, penciclovir, PGE2, tetracycline, uric acid, zidovudine
* SLC22A8*/OAT3	Adefovir, bile acids, cefaclor, ceftizoxime, cephaloridine, ciprofloxacin, conjugated sex hormones, methotrexate, NSAIDs, pravastatin, PGE2, uric acid, zidovudine
* SLC28A2*/CNT2	Adenosine, cladribine, clofarabine, didanosine, floxuridine, fluoropyrimidine, formycin B, guanosine, inosine, mizoribine, ribavirin, uridine, zidovudine
* SLC29A1*/ENT1	Adenosine, capecitabine, cladribine, cytarabine, cytosine, fialuridine, fludarabine, gemcitabine, guanine, guanosine, purine nucleosides, pyrimidine nucleosides, ribavirin, uridine, thymine, thymidine
* SLC29A2*/ENT2	2-Chloroadenosine, adenosine, cladribine, cytarabine, cytosine, fludarabine, formycin B, gemcitabine, guanine, guanosine, hypoxanthine, inosine, thymidine, thymine, tubercidin, uridine, vidarabine, zidovudine
* SLC51A*/OSTα	Bile acids, conjugated steroids, DHEAS, digoxin, PGE2
* SLC51B*/OSTβ	Bile acids, conjugated steroids, DHEAS, digoxin, PGE2
* SLCO2B1*/OATP2B1	Aliskiren, amiodarone, atorvastatin, bosentan, bromsulphthalein, dehydroepiandrosterone sulphate, estrone-3-sulphate, fexofenadine, glibenclamide, L-thyroxine (T4), talinolol, telmisartan

### Enterocyte transporter localization

**Fig. 1:**
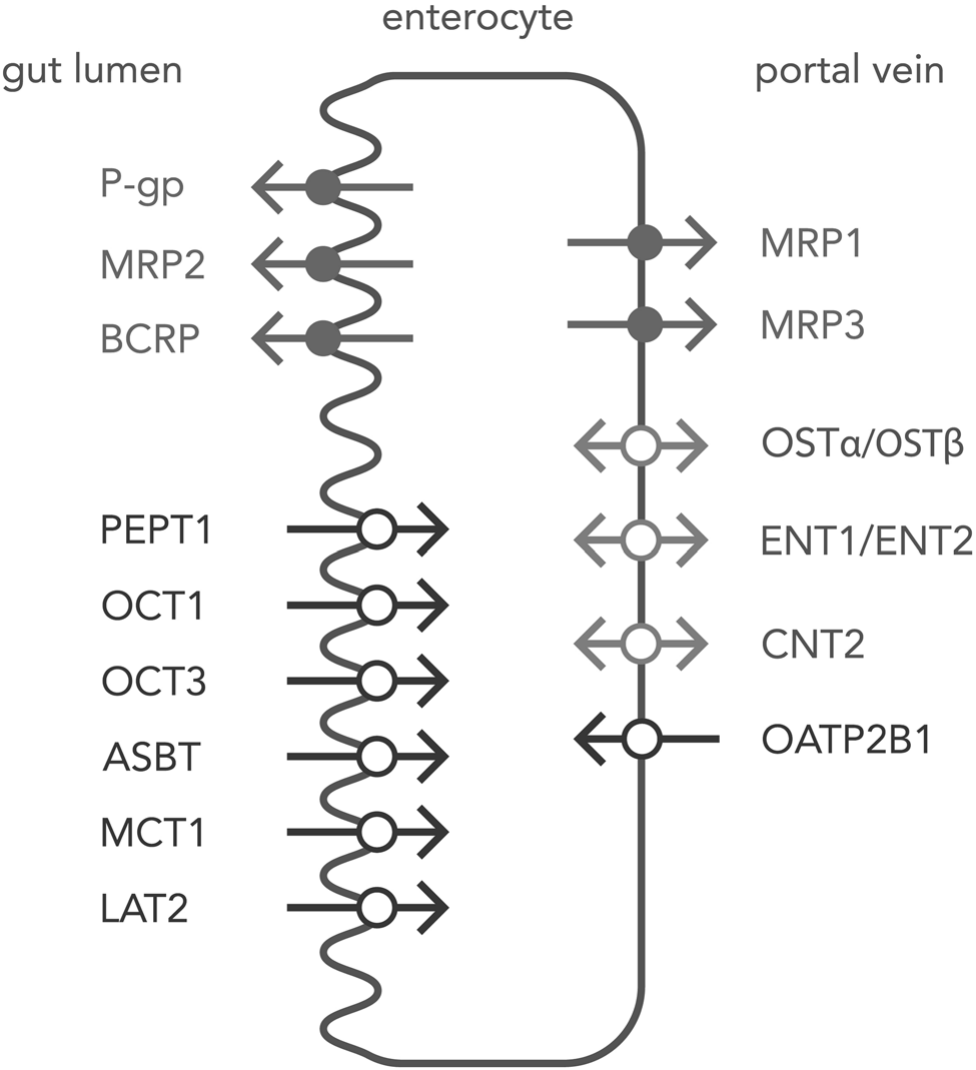
Enterocyte drug transporters

Enterocytes are endowed by a set of both ABC transporters and SLC carriers, which are localized in the apical and basolateral membranes [2, 3]. The enterocyte apical (luminal) membrane hosts uptake carriers, i.e., organic anion transporting polypeptides (OATP), organic cation transporter 1 (OCT1, *SLC22A1*) and 3 (OCT3, *SLC22A3*), peptide transporter 1 (PEPT1, *SLC15A1*), apical sodium-dependent bile acid transporter (ASBT, *SLC10A2*) and/or monocarboxylic acid transporter 1 (MCT1, *SLC16A1*) as well as efflux function transporters, i.e., ABCB1 (P-glycoprotein, MDR1, *ABCB1*), ABCC2 (MRP2) and breast cancer resistance protein (BCRP, *ABCG2*). The basolateral membrane of intestinal epithelia harbors heteromeric organic solute transporter functioning as bidirectional carrier (OSTα-OSTβ, SLC51), as well as MRP1 (*ABCC1*) and MRP3 (*ABCC3*) as uptake system or OATP2B1 (SLCO2B1) providing uptake activity. Some controversies exists as for membrane localization of OCT1 and OATP2B1, but the most recent findings provide evidence for the apical (OCT1) and basolateral (OATP2B1) expression (revised in Müller et al. [2]) (Fig. 1).

### Functional role of enterocyte transporters

The vectorial transport across the intestinal membrane affects drug absorption. Oral absorption of drugs is determined by intestinal ABC transporters providing efflux activity, such as P-gp, MRP2 or BCRP, which shuttle drug molecules back from enterocytes into intestinal lumen [4, 5]. On the contrary, the uptake carriers facilitate absorption of drugs, which possess structural similarity to their native substrates (e.g., β-lactam antibiotics, ACE inhibitors or antiviral drugs being peptide-like structures are substrates for the peptide carrier PEPT1) [6]. Also, it should be stated that the intestinal distribution of those transporters is not homogenous, and each segment of the gastrointestinal tract possess a specific set of transporters, that determine functional diversity, affecting, e.g., drug absorption. It seems that P-gp, ABCG2, PEPT1, and ASBT are more abundant in the jejunum and ileum than in the colon, in contrast the expression levels of ABCC2, ABCC3, and OCT3 are the highest in the colon [7, 8]. Therefore, absorption efficacy may depend on the intestinal segment where a drug is delivered, i.e., “absorption window”. Lower expression level of P-gp in the proximal small intestine stands behind more efficient absorption of its substrates in the upper intestine [9]. It is obvious, that not only intestinal drug transporters are unique determinants of transporter-mediated bioavailability, but a substantial contribution of hepatic transporter system function has to be highlighted, where a broad range of transporters is active, and in many cases higher abundant than in the intestinal tract. In humans, significantly higher hepatic protein content of MRP2, OCT1, and OATP2B1 were defined in reference to all intestinal segments. In the intestine, P-gp, BCRP and PEPT1 seem to play more important role in drug transport than in the liver [8].

The importance of intestinal P-gp function for drug pharmacokinetics was revealed in evaluation of the aforementioned drug “absorption window”. Clinical and experimental studies with talinolol and digoxin (P-gp substrates), by application of intestinal catheter perfusion or various dosage forms (immediate vs. extended release), demonstrated that drug delivery to distal parts of the small intestine produced markedly reduced drug bioavailability, most probably due to decreased absorption brought about by a higher P-gp-mediated efflux. Likewise, intestinal bypass or bowel surgery resulting in removal/circumvention of proximal part of the small intestine may involve substantially different oral drug absorption (including P-gp substrates) compared to the normal length gut (revised in Müller et al. [2]). It was also documented that drug-drug interactions (DDIs) for some statins are mediated by intestinal rather than hepatic BCRP inhibition. BCRP inhibition in the small intestine resulting in increased drug absorption is more likely than hepatic transporter function, as evidenced by changes in pharmacokinetic parameters. The intestinal contribution has higher potential to affect plasma area under the curve (AUC) and peak plasma concentration (C_max_), whereas hepatic/biliary BCRP downregulation may mainly produce increased drug hepatocyte exposure, since most statins are characterized by a relatively low membrane permeability, and thus are in need to use transporters to cross hepatocyte cell membranes [10]. This hypothesis can also be supported by physiologically-based pharmacokinetic modeling (PBPK) (combining in vitro and in vivo parameters) of pravastatin in rats for hepatic uptake and canalicular efflux [11]. PEPT1 is abundant in the gastrointestinal tract, but not in the liver, and effects on its expression and function may impact drug absorption. PEPT1 mediate transport of di/tripeptides and peptide-like drugs, such as ACE inhibitors or β-lactam antibiotics, increasing their oral bioavailability. Linking poorly absorbable molecules to structural motifs recognized by PEPT1 creates a kind of Trojan horse, which provides more effective absorption [12]. Valacyclovir (L-valine ester of acyclovir) characterized by highly improved bioavailability in comparison to acyclovir was first identified in the animal pharmacokinetic studies [13]. Afterwards, this strategy was used for development of several prodrugs to improve their oral bioavailability, such as oseltamivir or cefuroxim-axetil [6]. Therefore, the affected function of P-gp, BCRP and PEPT1, e.g., by pathological states, may be of special clinical significance.

Other transporters are mutually expressed in the liver and the gastrointestinal tract, and in this case the contribution of the gastrointestinal tract to the overall oral drug bioavailability may be less prominent (see below). However, there is some missing direct information about effects of intestinal transporters on drug pharmacokinetics, including pathology states.

Pathological states may directly affect transporter function in the gastrointestinal tract, when the process is active in intestines, e.g., inflammatory bowel disease. It was also evidenced that endogenous and exogenous (e.g., uremic toxins produced by intestinal bacteria) compounds generated during pathological processes can alter transporter state in intestines. This hypothesis was the best developed in the case of kidney failure, and is defined as remote sensing and signaling. As with other homeostatic systems, the drug transporters network, including the gastrointestinal system, promotes recovery of the organism or compensation for disturbances, i.e., in the case of kidney failure to eliminate uremic toxins [14].

The detained information about impact of pathological states on drug transporters and carriers expression and function in the gastrointestinal tract included in the review is presented in Table 2.

**Table 2:** Summary of drug transporter studies in pathological states

Transporter	Model/patients	mRNA	Protein	Function	References
Kidney failure					
* ABCB1*/P-gp	Caco2, IS, CMPF	–	–	↓ Cyclosporine transport	[18]
* ABCB1*/P-gp	Caco2, plasma from CRF rats	–	–	↓ 1.5-fold Rho123 transport	[15]
* Mdr1*/P-gp	Enterocytes, rat	–	↓ Twofold	–	[17]
* ABCB1*/P-gp	Caco2, plasma from CRF rats	–	–	↓ 1.4-fold Rho123 transport	[16]
* Mdr1*/P-gp	Enterocytes, rat, plasma from CRF rats	–	↓ 1.3-fold, WB	–	[16]
* Mdr1*/P-gp	Rat, gentamycin induced ARF	–	–	↓ 1.4-fold Rho123 exsorption clearance ↓ 1.1-fold cyclosporine A exsorption clearance, duodenal loop	[18]
* Mdr1a*/P-gp	Rat, 7/8 nephrectomy	↔ Jejunum, cPCR	↔ Intestine, ELISA	↓ Rho123 transport, everted gut sac	[20]
* Mdr1a*/P-gp	Rat, 5/6 nephrectomy	↔ Intestine, qRT-PCR	↓ 2.8-fold, intestine, WB	↓ 1.4-fold, Rho123 transport, everted gut	[16]
* Mdr1a*/P-gp	Rat, cisplatin induced ARF	–	–	↑ 1.3-fold tacrolimus bioavailability, ↑ 1.8-fold tacrolimus AUC_0–30_ in situ loop method—upper intestine	[21]
* ABCC2*/MRP2	Caco2, plasma from CRF rats	–	–	↓ 1.3-fold, CDF transport	[16]
* ABCC2*/MRP2	Enterocytes, rat, plasma from CRF rats	–	↓ 1.3-fold, WB	–	[16]
* Abcc2*/Mrp2	Rat, 5/6 nephrectomy	↔ Intestine, qRT-PCR	↓ 2.5 fold, intestine, WB	↓ 1.3-fold, CDF transport, everted gut	[16]
* Abcc3*/Mrp3	Rat, 5/6 nephrectomy	↔ Ileum, qRT-PCR	↔ Ileum, WB	–	[26]
* Abcg2/Bcrp*	Rat, 5/6 nephrectomy	↔ Duodenum, ↔ Jejunum, ↑ 1.5-fold, ileum, ↔ Colon, qRT-PCR	–	–	[22]
* Slco1a5*/Oatp1a5	Enterocytes, rat	–	↔	*–*	[17]
* Slco2*/Oatp2	Rat, 5/6 nephrectomy	–	↔ Intestine, WB	*–*	[16]
* Slco3*/Oatp3	Rat, 5/6 nephrectomy	↔ Intestine, qRT-PCR	↔ Intestine, WB	*–*	[16]
Slc10a2/Asbt	Rat, 5/6 nephrectomy	↔ Ileum, qRT-PCR	↔ Ileum, WB		[26]
* Slc15a1*/Pept1	Rat, 5/6 nephrectomy	↔ Duodenum ↔ Jejunum ↔ Ileum, cPCR	↑ Duodenum ↔ Jejunum ↔ Ileum, WB	↑ 2.3-fold AUC glycylsarcosine ↑ 1.8-fold AUC ceftibuten	[27]
* Slc51a*/OSTα	Rat, 5/6 nephrectomy	↔ Ileum, qRT-PCR	↔ Ileum, WB	–	[26]
* Slc51b*/Ostβ	Rat, 5/6 nephrectomy	↔ Ileum, qRT-PCR	↔ Ileum, WB	–	[26]
Liver failure					
* Abcb1*/Mdr1	Caco2, plasma from ALF rats	–	–	↓ 1.4-fold rhodamine transport	[32]
* Abcb1*/Mdr1	Caco2, plasma from ALF rats	–	–	↓ 1.3-fold rhodamine transport	[28]
* Abcb1*/Mdr1	Rat, ALF, thioacetamide	–	↑ 2.9-fold, intestine, WB	↑ 1.6-fold AUC, zidovudine, ↑ 1.7-fold, accumulative absorption, rhodamine 123	[30]
* Abcb1*/Mdr1	Rat, ALF, carbon tetrachloride	–	↔ Lower intestine, WB	↑ 3.4-fold digoxin absorption in vivo, ↓ 1.7-fold digoxin exsorption in everted intestinal sac	[29]
* Abcg2*/Bcrp	Rat, ALF, thioacetamide	–	↔ Intestine, WB	–	[30]
* Abcc2*/Mrp2	Rat, ALF, carbon tetrachloride	–	↓ 2.4-fold jejunum, WB	↓ 3.3-fold, DNP-GSH transport in everted intestinal sac	[31]
Cholestasis					
* Abcb1a*/Mdr1a	Mice C57BL/6, BDL		↔ , Ileum, WB		[33]
* Abcg2*/Bcrp	Mice C57BL/6, BDL		↑ 1.6-fold, ileum, WB		[33]
* Slc10a2*/Asbt	Mice C57BL/6, BDL		↔ , Ileum, WB		[33]
* Slc51a*/OSTα	Mice C57BL/6, BDL		↓ 1.7-fold, ileum, WB		[33]
* Slc51b*/Ostβ	Mice C57BL/6, BDL		↓ 5.0-fold, ileum, WB		[33]
Hyperthyroidism					
* ABCB1*/P-gp	Caco2, T4	↑ 5.5-fold, RT-PCR	↑ WB		[35]
* ABCB1*/P-gp	LS180V, T4	↑ 25.0-fold, RT-PCR			[35]
* ABCB1*/P-gp	Caco2, T3	↑ 10.0-fold, cPCR	↑ 2.5-fold, WB	↑ Tenfold transcellular transport, digoxin	[34]
mdr1a/mdr1b	Rat, T4	↔ Jejunum, ileum, cPCR	↔ Jejunum, ileum, WB	–	[36]
mdr1a/mdr1b	Rat, T4 (8 µg/kg, p.o., 3 weeks)	↑ 2.0-fold, ↔ Jejunum, ↔ Ileum, RT-PCR	↑ 2.0-fold, ↔ Jejunum, ↔ Ileum, WB	↔ PK, cyclosporin A (10 mg/kg, i.v.) ↓ Fivefold AUC, cyclosporin A (10 mg/kg, p.o.)	[37]
* ABCB1*/P-gp	Human (immunotherapy patients), T4 (100 µg, p.o., over 3 months)	–	–	Significant ↓ in cyclosporin A (p.o.) blood concentrations	[37]
* ABCB1*/P-gp	Human (volunteers), T4 (200 µg, p.o., 17 days)	↑ 1.4-fold, duodenum, cPCR	↑ 3.8-fold, duodenum, ICH	↔ PK, talinolol (30 mg i.v., 100 mg, p.o.)	[38]
* SLC15A1*/PEPT1	Caco2, T3	↓ 4.0-fold, cPCR	↓ 3.5-fold, WB	↓ Twofold Vmax, [14C]glycylsarcosine	[39]
* Slc15a1*/Pept1	Rat, T4	↓ 1.4-fold, cPCR	↓ 1.4-fold, WB	↓ 1.2-fold [14C]glycylsarcosine transport, intestinal loop ↓ 1.9-fold Vmax of [14C]glycylsarcosine	[40]
Hyperparathyroidism					
* ABCG2*/BCRP	Caco2, PTH (1–34)	↔ RT-PCR	↓ 1.3-fold, plasma membrane, WB, ICH, FC	–	[41]
* Abcg2*/Bcrp	Rat, SHPT	–	↔ Tissue homogenate, intestine, WB ↓, Plasma membrane, intestine, WB	–	[41]
Cushing syndrome (iatrogenic)					
* ABCB1*/P-gp	Rat, dexamethasone	↓ 2.0-fold, intestine, qPCR	↓ 1.3-fold, intestine, WB	–	[32]
* ABCC4*/MRP4	HT-29, prednisone	↑ 14-fold, RT-PCR	–	–	[42]
* ABCC4*/MRP4	HT-29, hydrocortisone	↔ , RT-PCR	–	–	[42]
Diabetes mellitus					
* ABCB1*/P-gp	Mice ddY, STZ	–	↓ 2.0-fold, ileum, WB	↓ 1.3-fold Rho123 excreting activity	[44]
* ABCB1*/P-gp	Mice ddY, STZ	–	↓ 2.5-fold, duodenum ↓ 3.0-fold, jejunum ↓ 1.7-fold, ileum, WB	–	[43]
* ABCB1*/P-gp	Mice ddY, MSG		↔ Duodenum, ileum; ↑ 2.2-fold, jejunum, WB		[43]
* ABCB1*/P-gp	Human, diabetes mellitus t.2	↔ Duodenum, RT-PCR	–	–	[45]
* ABCG2*/BCRP	Human, diabetes mellitus t.2	↔ Duodenum, RT-PCR	–	–	[45]
* SLC15A1*/PEPT1	Caco2, insulin	↔ cPCR	↑ 1.8-fold, membrane, WB	↑ 1.8-fold glycylglutamine transport	[46]
* SLCO2B1*/OATP2B1	Human, diabetes mellitus t.2	↑ 1.3-fold, duodenum, RT-PCR	–	–	[45]
Obesity					
* ABCB1/*P-gp	Rat, high-fat diet	–	↓ 1.2-fold, intestine, WB	↑ 1.2-fold nelfinavir bioavailability	[47]
* ABCB1/*P-gp	Human, obese	–	1.22 ± 0.37 fmol/μg protein, jejunum (LC–MS/MS)	–	[48]
* ABCC1*/MRP1	Human, obese	–	0.58 ± 0.20 fmol/μg protein, jejunum (LC–MS/MS)	–	[49]
* ABCC2*/MRP2	Human, obese	–	0.12 ± 0.04 fmol/μg protein, jejunum (LC–MS/MS)	–	[48]
* ABCC3*/MRP3	Human, obese	–	1.9 ± 0.74 fmol/μg protein (LC–MS/MS)	–	[48]
* ABCC4*/MRP4	Human, obese	–	0.31 ± 0.11 fmol/μg protein, jejunum (LC–MS/MS)	–	[49]
* ABCC5*/MRP5	Human, obese	–	0.05 ± 0.02 fmol/μg protein, jejunum (LC–MS/MS)	–	[49]
* ABCC6*/MRP6	Human, obese	–	0.30 ± 0.11 fmol/μg protein, jejunum (LC–MS/MS)	–	[49]
ABCG2/BCRP	Human, obese	–	1.25 ± 0.54fmol/μg protein, jejunum (LC–MS/MS)	–	[49]
* SLC7A8*/LAT2	Human, obese	–	5.09 ± 1.00 fmol/μg protein, jejunum (LC–MS/MS)	–	[49]
* SLC15A1*/PEPT1	Caco2, leptin	↔ cPCR	↑ 2.2-fold, membrane ↓ 2.0-fold, intracellular, WB	↑ 3.0-fold glycylsarcosine transport ↑ 2.2-fold cephalexin transport	[50]
* Slc15a1*/Pept1	Rat, leptin	–	–	↑ 1.5-fold cephalexin transport	[50]
* SLC15A1*/PEPT1	Human, obese	–	1.60 ± 0.71 fmol/μg protein, jejunum (LC–MS/MS)	–	[49]
* SLC16A1*/MCT1	Human, obese	–	1.85 ± 0.63 fmol/μg protein, jejunum (LC–MS/MS)	–	[49]
* SLC16A4*/MCT4	Human, obese	–	0.47 ± 0.10 fmol/μg protein, jejunum (LC–MS/MS)	–	[49]
* SLC22A4*/OCTN1	Human, obese	–	0.08 ± 0.01 fmol/μg protein, jejunum (LC–MS/MS)	–	[49]
* SLC28A2*/CNT2	Human, obese	–	0.84 ± 0.52 fmol/μg protein, jejunum (LC–MS/MS)	–	[49]
* SLC51A*/OSTα	Human, obese	–	4.45 ± 1.35 fmol/μg protein, jejunum (LC–MS/MS)	–	[49]
* SLC51B*/OSTβ	Human, obese	–	3.87 ± 1.02 fmol/μg protein, jejunum (LC–MS/MS)	–	[49]
* SLCO2A1*/OATP2A1	Human, obese	–	0.11 ± 0.05 fmol/μg protein, jejunum (LC–MS/MS)	–	[49]
SLCO2B1/OATP2B1	Human, obese	–	0.54 ± 0.13 fmol/μg protein, jejunum (LC–MS/MS)	–	[49]
General inflammation					
* ABCB1*/P-gp	Caco2, TNF-α, IFN-ɣ	↓ 2.0-fold, TNF-α ↑ 2.5-fold IFN-ɣ, cPCR	↑ 1.5-fold IFN-ɣ (24 h), WB	↓ Rhodamine 123 transport, TNF-α ↔ Rhodamine 123 transport, IFN-ɣ	[51]
* mdr1a*/P-gp	Rat, LPS	↓ 2.0-fold, duodenum ↓ 2.0-fold, jejunum ↓ 2.0-fold, ileum ↓ 2.0-fold, colon, RT-PCR	–	↓ 1.8-fold AUC_0–90_ min, digoxin, Ussig chamber	[53]
* mdr1a*/P-gp	Rat, adjuvant arthritis	↔ (RT-PCR)	–	–	[52]
* Abcc2*/mrp2	Rat, LPS	↓ 2.0-fold, jejumum, RT-PCR	–	↓ 2.7-fold AUC_0–90_ min, 5-carboxyfluorescein, Ussig chamber	[53]
* Abcc3*/mrp3	Rat, LPS	↔ RT-PCR	–	–	[53]
* ABCC4*/MRP4	HT-29, TNF-α, LPS	↑ Tenfold, TNF-α ↓ 3.3-fold, LPS, RT-PCR	–	–	[42]
* ABCC4*/MRP4	Human, intestinal tuberculosis	↓ 3.7-fold, RT-PCR	–	–	[42]
oatp1a1, oatp1b2, oatp2b1, oatp4a1, oat2, oat3, oct1, bsep, mrp1, mrp3, mrp6	Rat, adjuvant arthritis	↔ RT-PCR	–	–	[52]
Inflammatory bowel disease					
* ABCB1/*P-gp	Human, UC	↓ 2.9-fold, colon, qRT-PCR	↔ Colon, LC–MS/MS	–	[55]
* ABCB1/*P-gp	Human, IBD	↔ Colon (CD), ↓ 5.9-fold, colon (UC) qRT-PCR	–	–	[56]
* ABCB1/*P-gp	Human, UC	↓ 3.0-fold, sigmoid, qRT-PCR	↓ 7.8-fold, sigmoid, WB	–	[59]
* ABCB1/*P-gp	Human, UC	↓ 2.7-fold, colon, qRT-PCR	↓ 6.7-fold, colon, ICH	–	[58]
* ABCB1/*P-gp	Human, UC	↓ 1.2-fold, colon, ↓ 1.5-fold, rectum, qRT-PCR	↓ Colon, WB	–	[54]
* ABCB1/*P-gp	Human, UC	–	↑ 1.9-fold, colon, CD ↑ 1.4-fold, colon, UC, ICH	–	[57]
* ABCC1*/MRP1	Human, UC	↑ 1.6-fold, colon, qRT-PCR	↔ Colon, LC–MS/MS	–	[55]
* ABCC2*/MRP2	Human, UC	↑ 1.8-fold, sigmoid, qRT-PCR	–	–	[59]
ABCC2/MRP2	Human, UC	↔ Colon, qRT-PCR	–	–	[54]
* ABCC3*/MRP3	Human, UC	↔ Colon, qRT-PCR	↔ Colon, LC–MS/MS	–	[55]
* ABCC3*/MRP3	Human, IBD	↔ Colon (CD), ↓ 2.0-fold, colon (UC), qRT-PCR	–	–	[56]
ABCC4/MRP4	Human, UC	↑ 1.6-fold, colon, qRT-PCR	↔ Colon, LC–MS/MS	–	[55]
ABCC4/MRP4	Human, IBD	↓ 1.7-fold, colon (UC) qRT-PCR	–	–	[56]
ABCC4/MRP4	Human, UC	↓ 4.0-fold, colon, qRT-PCR	–	–	[42]
ABCG2/BCRP	Human, UC	↓ 5.6-fold, colon, qRT-PCR	BLQ	–	[55]
ABCG2/BCRP	Human, UC	↓ 1.8-fold, colon (CD), qRT-PCR	–	–	[56]
ABCG2/BCRP	Human, UC	↓ 2.8-fold, colon, qRT-PCR	↓ 2.9-fold, colon, ICH	–	[58]
ABCG2/BCRP	Human, UC	↓ 1.1-fold, colon, ↓ 1.2-fold, rectum, qRT-PCR	↓ Colon, WB	–	[54]
ABCG2/BCRP	Human, UC	↓ 1.5-fold, sigmoid, qRT-PCR	–	–	[59]
* SLC10A2*/ASBT	Human, UC	↓ 2.8-fold, colon (CD), qRT-PCR	–	–	[56]
* SLC15A1*/PEPT1	Human, IBD	↔ (CD), qRT-PCR	–	–	[67]
* SLC15A1*/PEPT1	Human, UC	↔ Colon, qRT-PCR	BLQ, LC–MS/MS	–	[55]
* SLC16A1*/MCT1	Human, IBD	↓ 2.0-fold, colon, qRT-PCR	↓ 3.9-fold, colon, LC–MS/MS		[55]
* SLC22A3*/OCT3	Human, IBD	↓ 4.3-fold, colon, qRT-PCR	BLQ, LC–MS/MS		[55]
* SLC22A4*/OCTN1	Human, IBD	↔ Ileum (CD), qRT-PCR	–	–	[67]
* SLC22A4*/OCTN1	Human, IBD	–	↔ Ileum, colon (CD), (WB)	↔ Carnitine transport (CD)	[68]
* SLC22A5*/OCTN2	Human, IBD	↓ 50.0-fold (CD), ileum, qRT-PCR,	–	–	[67]
* SLC22A5*/OCTN2	Human, IBD	–	↔ Ileum, colon (CD), (WB)	↔ Carnitine transport (CD)	[68]
* SLC22A5*/OCTN2	Human, UC	↔ Colon, qRT-PCR	BLQ, LC–MS/MS	–	[55]
* SLC28A2*/CNT2	Human, IBD	↔ Ileum (CD), qRT-PCR	–	–	[67]
* SLC29A1*/ENT1	Human, IBD	↑ 2.5-fold (CD), qRT-PCR	–	–	[67]
* SLC29A2*/ENT2	Human, IBD	↑ Fourfold (CD), qRT-PCR	–	–	[67]
* SLC51A*/OSTα	Human, IBD	↔ Colon (CD), ↓ 5.0-fold, colon (UC) qRT-PCR	–	–	[56]
* SLC51B*/OSTβ	Human, IBD	↔ Colon (CD), ↓ 3.4-fold, colon (UC) qRT-PCR	–	–	[56]
* SLCO1B1*/OATP1B1	Human, UC	↑ 1.7-fold, sigmoid, qRT-PCR	–	–	[59]
* SLCO1B3*/OATP1B3	Human, UC	↑ 15.8-fold, sigmoid, qRT-PCR	–	–	[59]
* SLCO2B1*/OATP2B1	Human, IBD	↑ Sevenfold (CD), ileum, qRT-PCR	–	–	[67]
* SLCO4A1*/OATP4A1	Human, IBD	↑ Fourfold (CD), ↔ (UC), ileum, qRT-PCR	–	–	[67]
Coeliac disease					
* ABCB1*/P-gp	Human, pediatric	↑ 12.0-fold (treated), ↑7.0-fold (untreated), qRT-PCR	–	–	[69]
Alzheimer disease					
* Abcc2*/mrp2	Mouse APP/PS1	–	↑ 2.3-fold, small intestine, QTAP	–	[70]
* Slc16a1*/mct1	Mouse APP/PS1	–	↓ 1.9-fold, small intestine, QTAP	–	[70]
* Slc7a8*/lat2	Mouse APP/PS1	–	↓ 2.0-fold, small intestine, QTAP	–	[70]

*ALF* acute liver failure, *ARF* acute renal failure, *BDL* bile duct ligation, *BLQ* below quantification range, *CD* Crohn disease, *CDF* 5-(and-6)-carboxy-2-,7-dichlorofluorescein, *CMPF* 3-carboxy-4-methyl-5-propyl-2-furanpropanoic acid, *IS* indoxyl sulfate, *cPCR* competitive PCR, *CRF* chronic renal failure, *DNP-GSH* 2,4-dinitrophenyl-S-glutathione, *FC* flow cytometry, *IBD* inflammatory bowel disease, *ICH* immunohistochemistry, *LPS* lipopolysaccharide, *MGS* monosodium glutamate, *PK* pharmacokinetics, *PTH* parathyroid hormone, *QTAP* quantitative targeted absolute proteomics, *SHPT* secondary parathyroidism, *STZ* streptozotocin, *T3* 3,5,3′-L-triiodothyronine, *T4* levothyroxine, *UC* ulcerative colitis, *WB* Western blot

## Kidney failure

Kidney failure, especially in advanced stage results in uremic toxin accumulation in plasma, which in turn affects function of multiple organs, including the gastrointestinal tract. In this regard, ABC transporters and SLC carriers are exposed to the action of the toxins, and according to the remote sensing and signaling hypothesis, mediate communication between organs and/or the host organism and gut microbiota [14].

### *ABCB1*/P-glycoprotein

Caco-2 cell (human intestinal epithelial cell line) studies demonstrated that plasma from the glycerol induced acute renal failure rats demonstrated more potent inhibitory effect on P-gp-mediated transport of rhodamine-123 (a model P-gp substrate) compared to plasma from healthy rats [15]. These observations were further confirmed by Naud et al. [16], who proved that uremic sera from 5/6 nephrectomized rats were demonstrated to induce a decrease in protein abundance and activity of P-gp compared with control sera measured in Caco-2 cells and rat enterocytes. Nolin et al. [17] confirmed the above observations using human serum from patients with end stage kidney disease. Exposure of rat enterocytes to the serum containing uremic toxins resulted to significant P-gp down-regulation. The authors postulated that uremic toxins, i.e., 3-carboxy-4-methyl-5-propyl- 2-furanpropanoic acid (CMPF) and indoxyl sulfate (IS) could be potential regulators of P-gp function. This hypothesis was confirmed by Shibata et al. [18], who revealed that both CMPF and IS inhibited P-gp mediated cyclosporine transport in Caco-2 cells. However, Sun et al. [19] were not able to reproduce those observations in MDR1–MDCK or Caco-2 cell study.

Animal models of chronic renal failure (CRF) confirm the observations from cell-based in vitro studies, providing evidence that kidney dysfunction modulates drug transporter activity in many organs, including the gastrointestinal tract. In studies with 7/8 nephrectomized rats (chronic renal failure model), Veau et al. [20] noted P-gp functional changes indicated by reduced rhodamine-123 transport in everted gut sacs from CRF rats. However, this study did not demonstrate significant changes in P-gp protein content and mRNA expression levels (*mdr1a*) in the jejunum. The quantitative changes in P-gp confirming reduced transport capacity of the intestine were observed in other studies. Decreased P-gp protein levels by more than 40% in the intestine was found by Naud et al. [16] in two-stage 5/6 nephrectomy induced CRF in rats, which were associated significant downregulation of P-gp activity (by 30%) in CRF rats compared with control. Likewise, to the aforementioned report, this study also demonstrated no effects of CRF on mRNA expression. Based on those observations it was postulated that the downregulation of P-gp in CRF depended on posttranslational processes. However, the mechanisms by which P-gp protein expression is reduced are to be determined. This study also demonstrated an inverse correlation between the creatinine clearance (and deteriorated kidney function) and P-gp protein abundance and activity. The findings suggest the presence of a molecule in serum responsible for these decreases. The findings of Shibata et al. [18] can provide an explanation for the observations related to decreased P-gp function in CRF. The authors revealed that P-gp-mediated cyclosporine transport was reduced in Caco-2 cells exposed to uremic toxins CMPF and IS, whose accumulation increases in the course of kidney failure.

Acute renal failure (ARF), likewise chronic organ dysfunction, is associated with reduced P-gp function, which was reported by Shibata et al. [18]. This study showed significantly decreased amounts of cyclosporine A (P-gp substrate) exported into the intestinal perfusate. The values of rhodamine-123 as well as cyclosporine A clearance from blood to the intestine were also significantly decreased in ARF animals in comparison to control rats.

The observed expression and functional changes of intestinal levels of P-gp in kidney failure entail changes in drug pharmacokinetics of accepted by the transporter. Reduced efflux function of enterocyte P-gp transporter, and thus less efficient secretion of its substrates back to the intestinal lumen in kidney failure, may contribute to increased drug concentrations, apart from reduced P-gp mediated elimination through the kidney. Okabe et al. [21] using intraintestinal and intraportal administration of tacrolimus estimated contribution of intestinal P-gp to tacrolimus pharmacokinetics in kidney failure induced by intraperitoneal administration of cisplatin. The authors observed significantly increased, by about twofold, tacrolimus (P-gp substrate) concentrations in renal failure rats in comparison with normal rats. The study demonstrated significantly increased absorption rate of the drug in the intestine due to P-glycoprotein down-regulation in rats with renal dysfunction (intestinal tacrolimus metabolism was not altered). Those results suggest that the drug accelerated absorption rate in the intestine in renal dysfunction produces partial saturation of hepatic extraction, which may be one of the mechanisms of increased bioavailability of tacrolimus (overall the bioavailability increased by about 35%).

### *ABCG2*/BCRP

Two-thirds of body urate is normally eliminated via the kidney, while the remaining one-third is excreted through the intestines via BCRP. Changes in *ABCG2*/BCRP levels in gastrointestinal tract in the course of CRF may represent an adaptation mechanism serving for uric acid (uremic toxin) elimination when the kidney pathway is impaired. Animal studies demonstrated that *Abcg2* expression in the ileum of 5/6 nephrectomized/kidney failure (characterized by significant, twofold increase in creatinine serum concentrations and respective twofold decrease in creatinine clearance) rats was significantly increased. The transporter levels in duodenum, jejunum and transverse colon were not affected by kidney dysfunction [22]. These findings suggest that ABCG2 overexpression in the ileum in kidney failure may constitute a compensatory mechanism in the state when renal excretion of uric acid is decreased, and thus possibly contribute to an increased uric acid excretion via the intestine. This statement can be reinforced by the study in *Abcg2*-knockout mice demonstrating significant reduction in intestinal urate excretion produced by depletion of Abcg2 [23]. Human studies also support the animal findings, and provide evidence that dysfunctional *ABCG2* genotypes (SNP s4148157) may be significantly associated with elevated serum uric acid levels in patients with CKD of European ancestry [24]. Other *ABCG2* SNPs were shown to be of functional value related to uric acid handling/transport, and were associated with early onset hyperuricemia and gout in Japanese population (rs2728125, rs223114) [25].

### *Mrp*/MRPs

There is few information on multiple-drug resistance related proteins (MRPs) expression and function in the gastrointestinal tract in kidney failure. The study of Naud et al. [16] provided evidence that CRF (in 5/6 nephrectomy rats) involves significant reduction, by more than 40%, in Mrp2 and Mrp3 protein levels in the intestine. This study demonstrated also decreased by about 25% Mrp2-mediated transport of its substrate 5-(and-6)-carboxy-2-,7-dichlorofluorescein (CDF), evidenced in the everted gut transport study. Similar to *Mdr1a* expression, mRNA levels of *Mrp2* were not affected by CRF, and involvement of posttranslational modifications was postulated by the authors. Uremic sera induced a reduction in Mrp2 protein expression in rat enterocytes. A weak positive correlation between Mrp2 protein expression and creatinine clearance was observed. These findings suggest that the protein expression and activity of the transporters decrease with the progression of renal failure, and thus advocate the existence of a serum molecule responsible for these decreases.

Contrary information about Mrp3 mRNA and protein levels was reported by Gai et al. [26]. These authors revealed unchanged levels of the transporter in 5/6 nephrectomized (8 weeks from the procedure) rats with CRF (in contrast to *Abcc3*/Mrp3 increase in the liver).

### *Slc*/SLC

Function of Pept1 using its substrates, i.e., [^14^C]Gly-Sar and ceftibuten, was studied by Shimizu et al. [27] in 5/6 nephrectomized rats. An increased intestinal carrier function associated with significant upregulation of Pept1 protein in the gastrointestinal tract in chronic renal failure animals was found. At the early stage of kidney dysfunction higher levels of Pept1 were seen in the duodenum, which further expanded to the jejunum in the course of chronic renal failure progression. In contrast, the expression in the ileum was similar to that in sham operated rats (till end of week 8 from surgery). The observed upregulation of Pept1 protein abundance was not associated with changes in mRNA levels. Those findings suggest, that enhanced bioavailability of oligopeptides determined by the PEPT1 enterocyte content may not only affect pharmacokinetics of its drug substrates, but also could be partially a risk factor for progression of chronic renal failure related to the dietary protein exposure.

Bile acid carriers, i.e., Asbt, Osta and Ostb, in the intestines seem to be not altered in the course of kidney failure. Gai et al. [26] documented that mRNA and protein levels Asbt, Osta and Ostb in ileum were not changed in 5/6 nephrectomized (8 weeks from the procedure) rats with CRF, in contrast to the alterations in bile acid transporter expression profile observed in hepatocytes.

### *Slco*/OATP

There is also an information about changes of Oatp1a5 protein content in normal rat enterocytes subjected to serum from patients with end stage renal disease for 48 h [17]. The levels of the transporter were not changed. Likewise, protein levels other Oatp transporters, i.e., Oatp2 and Oatp3 were not altered in rats with chronic renal failure resulting from two-stage 5/6 nephrectomy. The authors also provided information on mRNA expression for Oatp3, which was not changed in rats with CRF.

The molecular mechanisms triggering the transporter changes in the gastrointestinal tract remain to be elucidated. However, there is some information about possible involvement of endothelin 1 (ET-1) via its basolateral type B receptor (ET_B_) and through protein kinase C (PKC) as an intracellular second messenger system, which may negatively regulate MRP2 and P-gp in enterocytes (enterocytes express ET_B_ receptors, and increased endothelin-1 excretion is observed in CRF rats) [19]. Several studies have documented that CRF was associated with chronic inflammation, and cytokines could be considered as potential uremic mediators. The effects of cytokines on drug transporter expression and function was demonstrated (see below), resulting in functional downregulation.

## Liver failure

Likewise in the case of renal failure, changes in drug transporters in the gastrointestinal tract observed in liver dysfunction may represent adaptation mechanisms involved in bile acid and bile salts handling, and support the remote sensing and signaling hypothesis.

### *Abcb1*/P-glycoprotein

In vitro studies demonstrated that plasma from rats with acute liver failure (ALF) induced by administration of carbon tetrachloride (CCl_4_) exhibited significantly greater inhibitory potency on P-gp-mediated rhodamine-123 transport across Caco-2 cell monolayers than plasma from control rats. Similar effects on P-gp functions associated with exposure of Caco-2 cells to plasma form ALF rats (induced by intraperitoneal exposure to CCl_4_) were reported by Huang et al. [28]. Plasma from CCl_4_-treated rats produced more potent inhibitory effect on P-gp-protein-mediated shuttle of rhodamine-123 in comparison to the one from control animals, suggesting the presence of an endogenous P-glycoprotein substrate/inhibitor in the plasma of ALF rats. In fact, the authors identified that corticosterone (an endogenous P-glycoprotein substrate) plasma levels, were elevated twofold in CCl_4_-treated rats as compared with control animals, thus can interfere with the transporter functions.

Animal studies, i.e., carbon tetrachloride-induced ALF in rats, partially confirms the abovementioned findings in Caco-2 cell cultures. Western blot analysis demonstrated unchanged abundance of intestinal P-gp in ALF. However, the intestinal P-gp function in vivo was found to be significantly reduced in ALF, as studied by the P-gp absorption and exsorption substrates, i.e., digoxin and rhodamine-123. In contrast, P-gp function in vitro was significantly higher in ALF, as demonstrated by the efflux transport rate of P-gp substrates through the everted intestine. Therefore, the intestinal P-gp function was found to be differently affected by ALF in in vivo and in vitro conditions [29]. Another experimental model of ALF, i.e., thioacetamide-induced liver failure, confirms observations from CCl_4_-treated rats, as significant downregulation of intestinal P-gp expression was observed. The study provided also functional confirmation of the observed changes in transporter level, since ALF significantly elevated rhodamine-123 intestinal absorption, without affecting intestinal integrity in in vivo study, which points out on deterioration of intestinal P-gp function [30].

### *Abcg2*/BCRP

Experimental studies in rats with thioacetamide-induced ALF revealed mild, 1.6-fold (statistically not significant) upregulation of BCRP in liver failure [30].

### *Mrp*/MRPs

The expression and function of Mrp2 in the intestine was analyzed in CCl_4_-induced ALF rats with hyperbilirubinemia by Yokooji et al. [31]. It was found that Mrp2 protein levels in the jejunum were decreased to 41% of the controls abundance. However, impact of the liver failure on Mrp2 protein abundance was not observed in the ileum. The changes of the transporter protein abundance were associated with reduced (to 31% of controls) Mrp2-mediated efflux of its substrate, i.e., 2,4-dinitrophenyl-S-glutathione (DNP-GSH) in the jejunum in vitro. The transporter changes in vivo were almost completely downregulated, i.e., to the same level produced by probenecid (Mrp2 inhibitor). On the contrary to the jejunum, CCl_4_ treatment did not affect the efflux rate in ileum, and effects of probenecid on the efflux rate of DNP-GSH in control and acute hepatic failure states were not observed. These findings suggest that Mrp2 function is inhibited in ALF in the jejunum, where Mrp2 is highly expressed, but not in the ileum.

The mechanisms modulating drug transporter expression in ALF remain to be elucidated. Some observations suggest that corticosterone may be involved in P-gp downregulation [32], but other active components of plasma from ALF animals could also provide inhibitory P-gp effects, as it was evidenced by Murakami et al. [32] and Huang et al. [28].

## Cholestasis

### *Abcb1*/P-glycoprotein

Mdr1 protein abundance in the rat ileum was not affected by mechanical cholestasis induced by bile duct ligation (BDL) during 7 days post-surgery [33].

### *Abcg2*/Bcrp

Animal study reported by Mennone et al. [33] suggest that intestinal changes in Bcrp levels may contribute to the adaptive response to cholestasis. The authors found that BDL, which is an experimental model of obstructive cholestasis, resulted in significant increase in Bcrp protein expression in ileum by 7 days. Bcrp transporter mediates bile acid excretion into the gastrointestinal tract, and thus in cholestatic states, its increased intestinal function may provide an adaptive mechanism protecting from excessive systemic bile acids accumulation/exposure (levels of Bcrp in the liver and kidneys were downregulated by BDL in mice), and as postulated by the authors, may participate in handling of as yet to be identified substrates.

### *Slc*/Slc

The BDL model of mechanical cholestasis revealed also significant down-regulation of both Osta and Ostb protein levels in the gastrointestinal tract. The study also documented an elevation, although statistically not significant, in protein abundance of Asbt in the ileum following BDL [33]. These observations are in keeping with farnesoid X receptor-mediated feedback expression regulation of these transporters by bile acids. It was demonstrated that FXR activation by bile acids reduced the expression of ASBT, which mediates bile acid transport from the intestinal lumen into the enterocytes, whereas it increased the expression of OSTs, thus eliciting intracellular trafficking of bile salts from the apical to the basolateral membrane. The observed changes in Asbt, Osta and Ostb document adaptive mechanisms in enterocytes to the absence of bile acids produced by BDL.

## Hyperthyroidism

Thyroid hormones produce significant effects on development, structure, and function of the gastrointestinal tract, thus thyroid gland pathology may also affect the expression, protein abundance and activity of drug transporters.

### *ABCB1*/P-glycoprotein

Caco-2 cell model evidenced that 3,5,3′-L-triiodothyronine (T3) can regulate expression and function of P-gp. T3 treatment resulted in a concentration-dependent increase in *ABCB1* gene expression. The upregulated expression of the transporter gene resulted in significantly diminished [^3^H]digoxin (P-gp substrate) accumulation in Caco-2 cell monolayers as compared with control cells. Changes in *ABCB1* expression level under T3 treatment were also associated with acceleration of [^3^H]digoxin basal-to-apical transcellular transport [34]. Mitin et al. [35] provided similar information about upregulation of the expression of P-gp gene coding mRNA and transporter protein not only in Caco-2, but also LS180 cells (human colon carcinoma cell line) treated with levothyroxine (T4) and T3 in a concentration- dependent manner. The latter study provided also evidence that pregnane X receptor (PXR) was not engaged in modulatory effects of the hormones.

Studies in hyperthyroid rats suggest that thyroid hormones may regulate expression of mdr1a/1b in intestines (jejunum, ileum), although the effect was less prominent than in the liver and kidneys. *mdr1a*/*1b* mRNA levels were not increased in the jejunum and ileum of hyperthyroid rats (likewise in the liver, but contrary to significant increase in the kidney). Significant upregulation was only observed in the case of *mdr1b* gene in the ileum of rats exposed to T4. Western blot analysis revealed slight P-gp protein increase in the jejunum and ileum (but marked in the kidney and liver) in hyperthyroid animals [36]. It was also reported that levothyroxine administration over 3 weeks was associated with twofold significant increase in duodenal mRNA and protein levels of mdr1a and mdr1b in Wistar rats. Administration of T4 did not induce mdr1a and mdr1b changes in the jejunum and ileum. This study also provided functional verification of the observed quantitative changes in mdr1a and mdr1b levels in the duodenum, as decreased oral bioavailability of cyclosporine A was observed in rats [37].

Siegmund et al. [38] provided data from human study on healthy individuals under the age of 30 years. Oral levothyroxine administration tended to induce elevation of *ABCB1* mRNA expression and P-gp protein abundance in healthy volunteers. However, the observed changes did not involve significant alterations in talinolol (P-gp substrate) pharmacokinetics, most probably due to doses of levothyroxine not producing thyrotoxicosis (200 µg orally for 17 days). Observations from clinical studies may also suggest upregulation of intestinal P-gp levels. Jin et al. [37] revealed that long-term administration of levothyroxine (over 3 months) to patients administered cyclosporine A for immunosuppression resulted in significantly lower blood concentrations of the drug.

### *SLC*/SLC

Studies on the Caco-2 cell line have demonstrated that thyroid hormone T3 decreased PEPT1 carrier activity via transcription inhibition and/or decreased stability of PEPT1 protein and mRNA. The thyroid gland hormone produced a significant decrease in the amount of *SLC15A1* mRNA (25% of the control), paralleled by 70% PEPT1 protein amount decrease (of the control) in the apical membrane. Kinetic analysis revealed that T3 treatment involved significant decrease of [^14^C]glycylsarcosine (PEPT1 substrate) maximum uptake (V_max_) value but produced no effect on K_m_ value [39].

Animal studies in rats with hyperthyroidism induced by administration of L-thyroxine confirmed in vitro observations. Hyperthyroidism brought about a significant reduction in Pept1 mRNA expression and protein abundance levels in the small intestine. The Pept1 carrier activity, assessed using everted small intestinal tissues and in situ intestinal loop techniques, was demonstrated to be significantly decreased as measured by [^14^C]glycylsarcosine in hyperthyroid rats. Kinetic analysis showed that the [^14^C]glycylsarcosine uptake (V_max_) value was markedly reduced in hyperthyroid animals, whereas K_m_ value was not affected. Thus, functional studies confirm quantitative Pept1 results, suggesting the transporter downregulation in hyperthyroidism [40].

## Hyperparathyroidism

### *Abcg2*/Bcrp

Kidney dysfunction entails secondary parathyroidism, and increased levels of parathyroid hormone (PTH) seem to play a crucial role in regulation of the major urate transporter in the gastrointestinal tract, i.e., BCRP, leading to clinically observed hyperuricemia. Sugimoto et al. [41] demonstrated that incubation of Caco-2 cells with a PTH active derivative [PTH (1–34)] markedly reduced BCRP plasma membrane expression in a dose-dependent manner as evidenced by flow cytometry, immunocytochemistry and Western blot. However, the incubation with PTH did not influence *ABCG2* mRNA expression in Caco-2 cells, which suggested that BCRP changes in plasma membrane levels resulted from an alteration in the transporter protein localization, but not from the suppression of its synthesis, i.e., involvement of posttranscriptional mechanisms acting via PTH receptors.

The animal model of secondary hyperparathyroidism (SHPT), i.e., 5/6 renal nephrectomized rats fed with a high-phosphorus diet (P 1.2%, Ca 0.6%), demonstrated downregulation of the plasma membrane fractions of Bcrp. The changes in Bcrp membrane levels were abolished by administration of cinacalcet (calcimimetic agent, a PTH suppressor) [41].

The regulatory mechanisms triggering changes in BCRP levels are not entirely defined, but experimental observations propose that the cAMP-PI3K-Akt signaling pathway could contribute to the PTH-induced suppression of BCRP plasma membrane abundance.

## Cushing syndrome (iatrogenic)

### *Abcb1*/P-glycoprotein

Murakami et al. [32] revealed, that administration of dexamethasone increased P-gp levels in the intestine, which was paralleled by increased P-gp mediated exsorption of rhodamine-123 (approximately twofold).

### *ABCC4*/MRP4

In the HT-29 cellular (human colorectal adenocarcinoma) model Verma et al. [42] revealed that exposure to prednisolone caused a significant dose-dependent increase (up to 14-fold at a concentration of 500 µM) in *ABCC4* expression. Hydrocortisone in the concentration range from 10 to 500 µM did not affect *ABCC4* mRNA levels. This results suggest possible specific responses from *ABCC4* gene to different glucocorticoids.

## Diabetes mellitus

### *ABCB1*/P-glycoprotein

Animal models of diabetes mellitus, i.e., monosodium glutamate (MGG, type 2 diabetes model characterized by multiple metabolic abnormalities such as hyperinsulinemia, hyperglycemia or hyperlipidemia)- and streptozotocin (STZ, type 1 diabetes model, typical hyperglycemia model)-induced, provide evidence that not balanced glucose homeostasis might affect expression and function of drug transporters in the gastrointestinal tract, although the observations in these two models are not consistent. In 24 week-old MSG-treated mice, the duodenal P-gp expression tended to elevate, and significant increase was noted in the jejunum, but not in the ileum [43]. Another study of Nawa et al. [44] in mice STZ-induced diabetes model demonstrated contrary observations. A significant reduction in P-gp protein levels in the ileum were noted after STZ administration, and were associated with a remarkable decrease in P-gp function measured as rhodamine-123 excreting activity in an in situ closed loop model. The authors also demonstrated possible involvement of nitric oxide synthase activity in regulation of P-gp expression in diabetic conditions induced by STZ.

Human studies in patients with well-balanced glucose levels in diabetes mellitus type 2 subjects (with HbA1C levels of 7.2 ± 1.0%) (contrary to the abovementioned animal models with hyperglycemia and other metabolic diabetes-related alterations) demonstrated that the expression level of *ABCB1* was not affected by the disease [45].

### *ABCG2*/BCRP

The drug transporter expression study in duodenal biopsies in diabetes mellitus type 2 subjects with corrected glucose levels reported by Gravel et al. [45] revealed, that likewise to *ABCB1* expression, the *ABCG2* mRNA levels were comparable to healthy controls.

### *SLC*/SLC

Insulin itself, was demonstrated to directly regulate expression and function of membrane carriers. It was found, that exposure of Caco-2 cells to insulin at a physiological concentration resulted in no change in *SLC15A1* expression, but PEPT1 protein amount in the apical membrane promptly increased after the exposure. Changes in PEPT1 membrane abundance was associated with increased glycylglutamine (PEPT1 substrate) uptake by Caco-2 cells. PEPT1 membrane trafficking was suppressed by colchicine, which disrupts microtubules. These findings suggest that insulin triggers PEPT1 translocation from a preexisting cytoplasmic pool to cell membrane [46].

### *SLCO*/OATP

The only one transporter, whose gene expression was upregulated in human diabetes type 2 patients with defined mean HbA1C levels of 7.2% was *SLCO2B1*. Its levels were significantly 1.3-fold elevated. However the range of the observed changes is most probably of marginal clinical relevance [45]. The human studies on the expression of *ABCB1*, *ABCG2* and *SLCO2B1* suggest that correction of glucose levels is associated with normal transporter expression in the gastrointestinal tract. However, protein abundance was not measured, and constitute a missing gap of information.

## Obesity

It is known that obesity is characterized by a low‐grade inflammation in the adipose tissue, which produces and releases inflammatory mediators: cytokines (see below), adipokines and chemokines, that may affect transporter functions in many organs, including the gastrointestinal tract.

### *ABCB1*/P-glycoprotein

Animal studies on rats with obesity induced by a high-fat diet documented downregulation of intestinal P-gp (to 78% of controls) after 8-week high-fat feeding [47]. The observed changes in P-gp abundance in the intestines could explain an increased absorption of nelfinavir. The authors revealed a 1.2-fold increase in fraction of administered dose of the drug, which was able to reach systemic circulation (F) in the rats with a high-fat diet-induced obesity.

Jejunum mucosa expression of P-gp in morbidly obese volunteers, classified for a Roux-en-Y gastric bypass, was published by Lloret-Linares et al. [48]. The level of P-gp protein in intestinal mucosa of 1.22 ± 0.37 fmol/μg protein was reported. This study does not provide reference, normal values, but our P-gp data from donor tissue using the same analytical method (human intestinal/jejunum mucosa, membrane fraction, LC–MS/MS) revealed the protein levels of 0.66 ± 0.13 fmol/μg protein), and may suggest upregulation of the transporter abundance [7].

### *ABCC*/MRP

Expression of MRPs transporters in jejunum in morbidly obese volunteers revealed the following levels if the MRPs transporters protein abundance: MRP2 0.12 ± 0.04 fmol/μg protein and MRP3 1.91 ± 0.74 fmol/μg protein [47]. Likewise to P-gp it could only be possible to address these results to other studies using normal tissue. Comparison to our healthy organ donor studies (see above) it could be suggested that the MRP2 protein levels are downregulated (reference MRP2 0.78 ± 0.19 fmol/μg protein [7]), and MRP3 are upregulated (reference MRP3 0.58 ± 0.12 fmol/μg protein [7]) in obese subjects.

The above information was complemented by Miyauchi et al. [49], who reported drug transporter protein abundance (measured via liquid chromatography-tandem mass spectrometry) in human jejunum sampled from morbidly obese patients (details see in Table 2). This study also does not provide information about the transporters levels in healthy tissues, but comparison to our study (see above) suggest that the protein abundance of most studied transporters in obese patients is reduced vs. healthy controls (i.e., ABCC1, ABCC4, ABCC5, ABCC6, LAT2, MCT1, MCT4, CNT2, OCTN1, OSTα, OSTβ, OATP2A1), but ABCG2, PEPT1 and OATP2B1 protein levels were similar to healthy subjects [7].

### *SLC15A1*/PEPT1

It was found that leptin is able to increase membrane PEPT1 protein levels, reduce its intracellular content, and produce no change in *SLC15A1* mRNA levels in Caco-2 cells. The change in membrane carrier abundance was associated with increased Caco-2 cell transmembrane transport of PEPT1 substrates: cephalexin and glycylsarcosine, which was abrogated by colchicine, an agent disrupting protein translocation to plasma membrane. In vivo rat study confirmed functional changes observed in the cellular model, and demonstrated that leptin was able to induce a rapid, twofold increase in plasma cephalexin levels, following cephalexin jejunal perfusion rats, thus demonstrating enhanced intestinal absorption of the carrier substrate [50].

## General inflammation

### *ABCB1*/P-glycoprotein

A range of proinflammatory cytokines were studied in cellular models as potential inducers/suppressors of drug transporter expression. It was revealed that TNF-α induced a strong time-dependent reduction (− 56%) in *ABCB1* mRNA, which was confirmed in functional experiments showing a significant reduction in rhodamine-123 unidirectional transport after 48 h exposure time in Caco-2 cells. The confocal laser scanning microscopy revealed mainly P-gp apical plasma membrane localization in both control and TNF-α-treated cells. On the contrary, IFN-ɣ induced up-regulation of both mRNA *ABCB1* and P-gp protein expression (at 24 h, but then downregulated at 72 h) without effects on P-gp activity [51].

An animal model of chronic inflammation produced by exposure to *Mycobacterium butyricum* suggest that *mdr1a* mRNA expression in intestinal tissues was not significantly changed (may be due to a high variability of the results), but a strong trend towards downregulation was seen [52]. However, the endotoxin-treated rat study provides a clear evidence that infection and inflammatory diseases may impose marked changes in intestinal transporters, and revealed significant downregulation of *mdr1a* mRNA expression by approximately 50% in the duodenum, jejunum, ileum and colon of LPS-treated rats [53]. Functional observations from the Ussing chamber studies on jejunal segments were in line with the mRNA expression study, and significant reduction in the basolateral to apical efflux of digoxin (P-gp substrate) was found, resulting in marked increases in the apical to basolateral absorption of the compound. Mannitol permeability and lactate dehydrogenase release were not altered, thus confirming the integrity of the intestinal wall. In this study the jejunum level of *mdr1b* expression was low and not significantly changed.

### *Abcg2*/Bcrp

Likewise *mdr1a*, *bsep* mRNA expression in the intestine was not affected by chronic inflammatory process induced by exposure to *Mycobacterium butyricum* in rats [52].

### *ABCC*/MRP

Observations from another cellular model, i.e., HT-29 cells (human colon cancer cell line) revealed that TNF-α up-regulated *ABCC4* expression in a dose-dependent manner, up to tenfold at concentration of 100 mg/ml [42]. Reactive oxygen species (ROS) and NF-κB were proposed as mediators for this signaling, as ROS and NF-kB inhibitor (N-acetylcysteine) prevented this up-regulation. In the contrary to TNF-α, lipopolysaccharide (LPS) down-regulated up to 3.3-fold (at concentration of 100 mg/ml) *ABCC4* levels in a dose-dependent manner.

The observations from LPS (endotoxin)-treated rats demonstrated significant by approximately 50% downregulation of *mrp2* expression in the jejunum [53]. The Ussing chamber study revealed reduction in the basolateral to apical efflux of 5-carboxyfluorescein (mrp2 substrate), resulting in marked increase in the apical to basolateral absorption of the compound in the jejunum, thus providing functional evidence related to decreased mdr2 transporter levels. Mannitol permeability and lactate dehydrogenase release were not affected.

Animal studies on chronic inflammation (induced by *Mycobacterium butyricum* exposure) provided evidence that *mrp1*, *mrp3* and *mrp6* were not significantly affected by the pathological process [52]. The findings on *mrp3* expression were confirmed in LPS-treated rats, where similar *mrp3* levels to the controls in the jejunum were noted [53].

### *Slc*/SLC

Experimental model of chronic inflammation, i.e., exposure of rats to *Mycobacterium butyricum* revealed that systemic inflammatory state did not produce significant differences for mRNA expression of *oat2*, *oat3* and *oct1* in intestinal tissues. However, *oct1* expression demonstrated a strong trend towards downregulation [52].

Observation in human tissues also provide evidence, that inflammation within the gastrointestinal tract may trigger changes in drug carriers’ expression. Verma et al. [42] provided information about significant *ABCC4* mRNA down-regulation in inflamed intestinal regions in patients suffering from intestinal tuberculosis in comparison to healthy subjects.

### *Slco*/Oatp

The adjuvant arthritis induced by exposure to *Mycobacterium butyricum*, an animal model of chronic inflammation, was used to define the impact of inflammatory state on intestinal drug transporters, including *slco*/oatp carriers. Due to high variability of mRNA levels in intestinal tissue no significant differences were observed for expression of *oatp1a1*, *oatp1a5*, *oatp1b2*, *oatp2b1* and *oatp4a1* [52]. Infections and inflammatory diseases may affect drug bioavailability through effects produced on intestinal expression and activity of drug transporters.

## Inflammatory bowel disease

Inflammatory bowel disease (ulcerative colitis—UC and Crohn’s disease—CD) (IBD) should be considered not only by local intestinal inflammatory status, but also as general inflammation, as altered expression profile of drug transporters is seen in non-inflamed intestinal tissues and even outside of the gastrointestinal tract (e.g., in the liver).

### *ABCB1*/P-glycoprotein

Significantly reduced ABCB1 expression levels in UC patients with active inflammation in comparison with controls were seen by Englund et al. [54] and Erdmann et al. [55]. However, Jahnel et al. [56] reported that *ABCB1* levels did not differ significantly between controls and patients with active CD or UC in remission. In turn, in pancolitis in the course of UC, mRNA expression levels for *ABCB1* the basolateral efflux pumps were significantly reduced to 17% of normal colon mucosa values.

Plewka et al. [57] provided immune-quantitative protein information about P-gp and its cellular localization. The expression P-gp was found exclusively in the apical membranes of enterocytes from CD, UC and controls. In CD cases P-gp expression amounted to 190% and in UC to 145% of the control values. However, Jahnel et al. [56] likewise to Gutmann et al. [58] presented that unaffected mucosa in UC patients was characterized by lower mRNA levels of *ABCB1* in comparison with control subjects. The mRNA expression was associated with reduced P-gp staining (by 85%) in UC inflamed mucosa. Ufer et al. [59] complemented the information about P-gp in UC of moderate and severe symptomology, by a finding of a significant inverse correlation between *ABCB1* expression, P-gp protein abundance and clinical activity index in UC patients.

Altered levels of P-gp may affect the treatment outcome of IBD. Expression of P-gp may be involved in modulation of drug response to 5-amino salicylic acid, immunosuppressant drugs and corticosteroids [42, 60]. Gutmann et al. [58] evidenced that patients administered with 5-aminosalicylates and not responding to the medication demonstrated a marked suppression in P-gp expression in the inflamed mucosa in comparison with the unaffected tissue. On the other hand, patients subjected to prednisone revealed only unremarkable decrease of P-gp. However, some of glucocorticoids were defined to be substrates of P-gp, thus its altered status might affect drug response [61].

P-gp was also postulated to play a role in a pathophysiology of IBD. It was found that *mdr1a-*deficient mice are characterized by an ulcerative colitis-like phenotype, a state which was reversed with antibiotics [62]. Most probably mdr1 transporter provides defense mechanism against bacterial toxins damaging intestinal mucosa. Clinical studies (see above) suggest downregulation of *ABCB1*/P-gp in IBD, supporting the abovementioned hypothesis. These findings are supported also by studies, which suggest associations of *ABCB1* polymorphisms with UC susceptibility in humans. The *ABCB1* C3435T single nucleotide polymorphism associated with reduced expression of P-gp in the gastrointestinal tract was found in some populations of patients with extensive UC, but not all [63].

### *ABCG2*/BCRP

Information about another ABC-transporter, *ABCG2*/BCRP, was presented by Jahnel et al. [56], who found reduced *ABCG2* mRNA expression in inflamed regions in subjects with CD ileitis than in unaffected CD ileal mucosa. Likewise, Englund et al. [54] and Erdmann et al. [55] demonstrated marked reduction of *ABCG2* expression in UC patients with an active inflammation compared with healthy subjects. The BCRP positive staining of colon epithelium was reduced in inflamed/affected samples, with coexisting disruption of epithelial F-actin structure, as compared to healthy mucosa. This observation is consistent with the Gutmann et al. [58] study, that demonstrated markedly reduced BCRP staining (by 63%) in the inflamed mucosa of active UC patients. However, LC–MS/MS analysis of BCRP protein did not confirm its presence in UC non-inflamed and inflamed tissues as well as in controls [55]. However, in this case drug treatment should be taken into account when analyzing levels of the transporters.

Likewise to P-gp, BCRP levels were postulated to affect response to 5-amino salicylic acid, corticosteroids and immunosuppressants [42, 58, 60]. It was reported that patients treated with 5-aminosalicylates and not responding to the drug showed a considerable downregulation of BCRP in the inflamed mucosa (in comparison with the unaffected tissue). However, patients medicated with prednisone demonstrated only an insignificant decrease in the transporter level. BCRP was also suggested to mediate sulfasalazine absorption [64].

Similar to P-gp, BCRP providing efflux function in the apical membrane of enterocytes may play an important role in tissue defense against xenobiotics, including toxins and carcinogens [65, 66]. Those observations, i.e., decreased levels of the transporter in IBD, may support clinical findings documenting an increased risk of colon cancer as well as greater anatomic extent in long-term colitis.

### *ABCC*/MRP

Erdmann et al. [55] reported significant downregulation of *ABCC1* in inflamed mucosa in patients with UC, but mRNA changes were not accompanied with marked reduction in MRP1 protein abundance.

The expression of *ABCC2* in colon, likewise to *ABCC1*, seems to be not affected by the inflammatory processes associated with CD as reported by Englund et al. [54].

Jahnel et al. [56] reported that *ABCC3* expression did not differ significantly between healthy subjects and patients with active CD or UC in remission. In turn, in pancolitis in the course of UC *ABCC3* mRNA expression levels fell significantly to 50% of healthy colon mucosa values. However, comparable *ABCC3* mRNA expression and MRP3 protein levels in noninflamed and inflamed tissues in patients with UC as well as in controls were reported by Erdmann et al. [55].

Significantly increased levels of *ABCC4* mRNA in active UC vs. healthy subjects was observed by Verma et al. [42] and Erdmann et al. [55]. Contrary, Jahnel et al. [56] demonstrated markedly decreased levels of MRP4 (to 58% of controls) in UC patients. This observation was confirmed by LC–MS/MS analysis of MRP4 protein abundance in inflamed UC samples [55]. In patients with UC in remission no significant changes in *ABCC4* expression levels in comparison with mRNA levels in controls or in active UC patients were noted.

### *SLC*/SLC and *SLCO*/OATP

There is evidence, that IBD can also affect SLC cariers’ levels. Wojtal et al. [67] reported that intestinal tissues from patients diagnosed with UC and CD (analyzed together) were characterized by significantly elevated mRNA levels of *SLC29A1* (ENT1), SLC29A2 (ENT2), and OATP2B1, while ASBT and OCTN2 were significantly downregulated in the ileum of IBD patients. In the non-inflamed colon, mRNA levels for *SLC28A2*, *SLC29A1*, *SLC29A2*, *SLCO2B1*, and *SLCO4A1* were markedly upregulated, while mRNA level for *SLC22A5* (OCTN2) was significantly downregulated. In inflamed colon of IBD patients the mRNA levels of *SLC28A2* (CNT2), *SLC29A1*, *SLC29A2, SLCO2B1*, *SLCO4A1*, and *SLC15A1* (PEPT1) were significantly elevated. Likewise, Jahnel et al. [56] reported considerable decrease in *SLC10A2* (ASBT) expression levels in ileitis in inflamed areas in patients, yielding 36% of values in healthy-subject ileal mucosa. A less pronounced but also meaningful reduction was noted in CD subjects in remission, in whom ASBT expression levels yielded 53% of controls. However, it should be noted that LC–MS–MS analysis of UC tissues (both inflamed and non-inflamed) did not reveal protein abundance (below of quantification limit) of ASBT, OCT1, OCT3, OCTN2, PEPT1 in colon samples (likewise in control tissues). Therefore, the mRNA reports on UC-induced changes in transporter levels should be analyzed with caution. The same study reported stable protein levels of OATP2B1 in UC inflamed and non-inflamed as well as control tissues. The only protein significantly downregulated during UC (both inflamed and non-inflamed samples) was MCT-1 (also at mRNA level—*SLC16A1*) [55].

Protein information about OCTN1 and OCTN2 transporters in ileal and colon tissues sampled from patients with CD was reported by Girardin et al. [68]. The study revealed that OCTN protein levels appeared to be comparable in CD intestines and controls, measured by Western blot, but OCTN1 expression was higher in mutant homozygous or heterozygous genotype CD patients. Likewise, ileal carnitine transport was defined as very rapid and Na^+^-dependent, and to be similar in CD and healthy groups. A trend towards higher carnitine transport in subjects with OCTN1 and OCTN2 mutations was observed. The OCTN2 protein data is contrary to *SLC22A5* mRNA expression information reported by Wojtal et al. [67]. The data of Girardin et al. [68] also postulates an effect of genetic constitution on OCTN1 and OCTN2 activity.

SLC carriers might also participate in transport of drugs used in the treatment of IBD, namely mesalazin is an OATP2B1 substrate, therefore changes in the transporter function may modulate response to the drug [61].

## Coeliac disease

A scarce information about expression of the transporters in coeliac disease is available. Vannay et al. [69] observed comparable *ABCB1* mRNA expression in duodenal mucosa in pediatric patients with untreated celiac disease in comparison with healthy controls, with an increasing trend in the disease progression. Introduction of gluten-free diet precipitated significant raise in transporter expression in the duodenum of celiac disease patients (vs. controls and untreated patients).

## Alzheimer disease

Abundance of drug transporters in the small intestine in APPswe/PSEN1dE9 (APP/PS1) transgenic mice [widely-used Alzheimer disease model, expressing a chimeric mouse/human amyloid precursor protein (Mo/HuAPP695swe) as well as a mutant human presenilin 1 (PS1-dE9)] mice, measured via quantitative targeted absolute proteomics (QTAP) was evaluated by Pan et al. [70]. A significant 2.3-fold increase in Mrp2, 1.9-fold decrease in Mct1 and twofold decrease in large amino acid transporter 2 (Lat2) were observed. The levels of other studied transporters, i.e., Mdr1a, Mrp3, Mrp4, Mrp6, Bcrp, Abcg5, Abcg6, Mate1, Ostb, Oatp2b1 remained unchanged in comparison to the wild-type mice.

## Conclusions

The accumulated data suggest that both the gastrointestinal pathologies (inflammatory bowel disease, celiac disease, cholestasis), systemic pathology states (kidney failure, liver failure, hyperthyroidism, hyperparathyroidism, obesity, diabetes mellitus, systemic inflammation and Alzheimer disease) affect the expression and function of the drug transporters in the gastrointestinal tract. The altered status the transporters provide compensatory activity in handling endogenous compounds, affect local drug actions in the gastrointestinal tract as well as impact drug bioavailability. P-gp, BCRP and MRPs belong to the best defined transporters. However, most of the available information about drug transporters and carriers in the gastrointestinal tract during pathology states are derived from in vitro and/or animal studies, and should be verified in further clinical observations.
